# First report of *Tequus schrottkyi* (Konow) (Hymenoptera: Pergidae) in Uruguay, and information about its host plant and biology

**DOI:** 10.3897/BDJ.4.e7538

**Published:** 2016-01-13

**Authors:** Paula Altesor, Andrés González, Stefan Schmidt

**Affiliations:** ‡ Facultad de Agronomía, Universidad de la República, Montevideo, Uruguay; § Facultad de Química, Universidad de la República, Montevideo, Uruguay; | SNSB-Zoologische Staatssammlung München (ZSM), Munich, Germany

**Keywords:** *Tequus schrottkyi*, sawfly, Pergidae, Symphyta, *Solanum commersonii*, potato

## Abstract

**Background:**

The sawfly family Pergidae is best represented in South America, and it is the third largest family in the suborder Symphyta. *Tequus* is a Neotropical genus that has been reported in association with host plants of the genus *Solanum* (Solanaceae), with little information about the life history of its members. *Tequus schrottkyi* (Konow, 1906) was described from Paraguay, without any information about its biology and host plant.

**New information:**

We report the first record of *T. schrottkyi* from Uruguay, with information on its host plant and details of its biology. The identification was based on morphology, DNA barcode is provided to allow identification using molecular characters. This sawfly species is associated with *Solanum commersonii*, a native plant common in Uruguay. *Tequus schrottkyi* presents several generations between March and July. The larvae feed on leaves and spin a silk cocoon in the soil in which they pupate. The adults exhibit sexual dimorphism, the female being larger than the male and with a different color pattern. The eggs are laid individually in the leaf margins into the leaf tissue. The larvae are unpalatable to a generalist predator, possibly due to defensive compounds sequestered from their host plant, known to contain toxic compounds.

## Introduction

The sawfly family Pergidae is distributed in North and South America and Australasia, with the majority of species occurring in South America (Schmidt and Smith 2006). It is the third largest family of the suborder Symphyta, after the Tenthredinidae and the Argidae, with currently 12 subfamilies, 60 genera, and 441 described species (Schmidt and Smith 2015). For most species there is little or no information about their biology and the plants on which they feed as larvae (Schmidt and Smith 2006).

The genus *Tequus* occurs in the Neotropical region and includes 14 species that have been recorded from the following countries: Argentina, Bolivia, Chile, Colombia, Nicaragua, Paraguay and Peru (Schmidt and Smith 2015). Larvae of a few *Tequus* species have been found associated with plants of the genus *Solanum* (Solanaceae) (Schmidt and Smith 2015), and some species occurring in Peru and Bolivia are economically important because they feed on the cultivated potato, *S. tuberosum* (Carrasco 1967, Munro 1954, Wille 1943, recorded as *Acordulecera* spp.). As with the family in general, there is little information about the biology of *Tequus* species. A key to species (as *Acordulecera* spp.) was given by Smith 1980 who later proposed a new genus *Tequus* for the members of the species group (Smith 1990). The genus can be separated from *Acordulecera* by the following characters: head widened behind eyes, antenna and lower interocular distance longer than in *Acordulecera*, mesoscutellum with large flangelike carina and posterior margin of metascutellum carinate (Smith 1990). In addition, the female saw of most species shows some peculiarities that are characteristic for the genus (figs 487-496 in Smith 1990).

Here we report the first record of *Tequus schrottkyi* (Konow, 1906) from Uruguay, with information about its host plant and details about its biology. This species was originally described from Paraguay, but without any information about its host plant.

## Materials and methods

*Tequus schrottkyi* was found for the first time in a field experiment carried out in 2011 on the Experimental Station INIA - Las Brujas, Canelones, Uruguay (34°39’49.62"S; 56°20’23.23"W). This field plot experiment was carried out to compare the susceptibility of *Solanum tuberosum* and a native congener, *S. commersonii*, to insect herbivores. *Tequus schrottkyi* larvae were found almost exclusively feeding on leaves of *S. commersonii* (Altesor et al. 2014).

**Figure 1.**
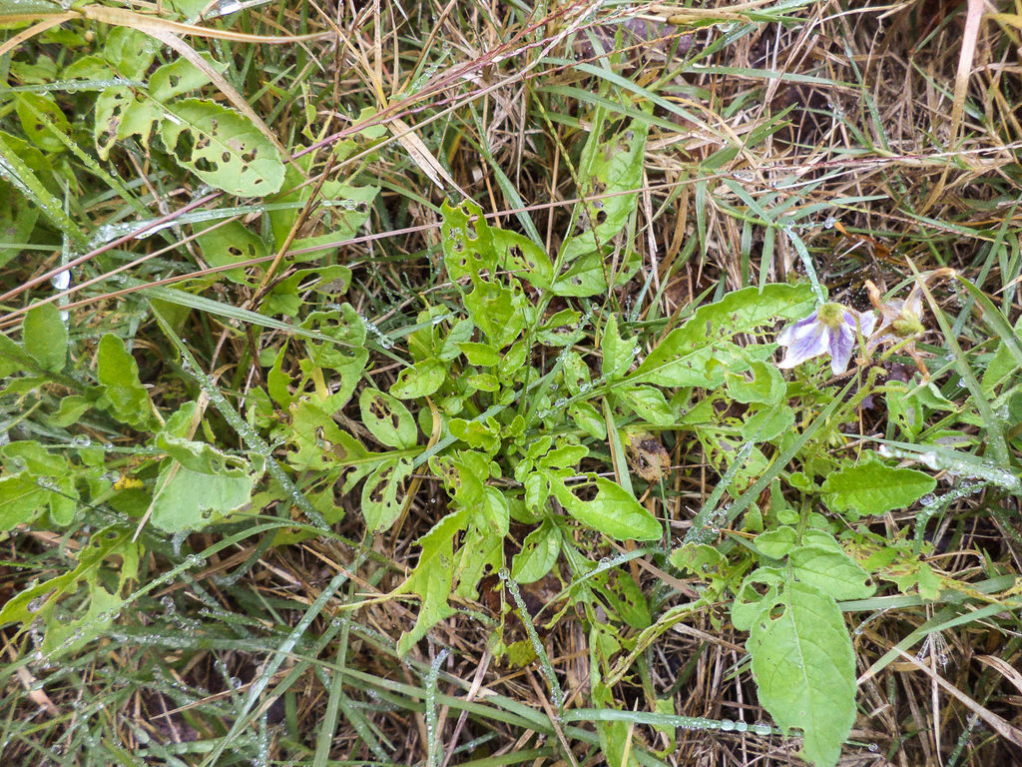
*Solanum commersonii* eaten by larvae of *T. schrottkyi* at INIA-Las Brujas field station.

Between 2013 and 2015, seasonal samples were taken of *S. commersonii* plants in INIA - Las Brujas to evaluate the presence of *T. schrottkyi* (Fig. 1). Eggs and larvae were taken to the laboratory to test their feeding preferences (Altesor et al. 2014) and growth performance (P. Altesor, unpublished) on both *Solanum* species, thereby obtaining information about its biology that is included in present report.

## Taxon treatments

### Tequus schrottkyi

(Konow 1906)

http://www.boldsystems.org/index.php/Public_SearchTerms?query=DS-TEQSCH

Tequus schrottkyi *Acorduleceros Schrottkyi*Konow 1906: 345-346. Type locality: Paraguay: Villa Encarnación. Lectotype female, designated by Smith 1980: 101. Type depository: Senckenberg Deutsches Entomologisches Institut, Müncheberg, Germany. Described: female.
Acordulecera schrottkyi : Smith 1978: 179.
Tequus schrottkyi : Smith 1990: 190.

#### Distribution

Paraguay, Uruguay

#### Ecology

In INIA - Las Brujas field station, *T. schrottkyi* larvae were only found feeding on *S. commersonii* in autumn and early winter between March and July. *Solanum commersonii* is a perennial plant that has its center of distribution in Uruguay, but also occurs in Paraguay, Brazil and Argentina (Spooner and Hijmans 2001). Therefore, this plant may also be the host of *T. schrottkyi* in Paraguay, where it was originally reported. Since *S. commersonii* foliage is less available during spring and summer in this area, it is assumed that *T. schrottkyi* enters diapause and/or moves to another host plant during the rest of the year.

**Figure 2.**
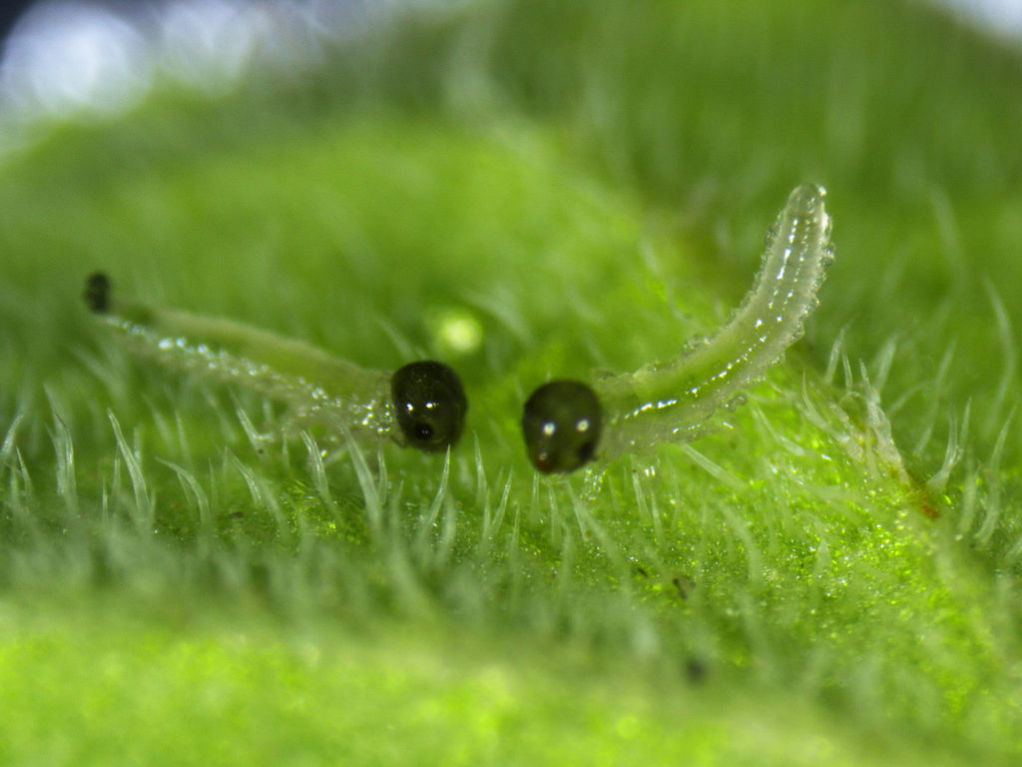
First instar larvae of *T. schrottkyi*.

**Figure 3.**
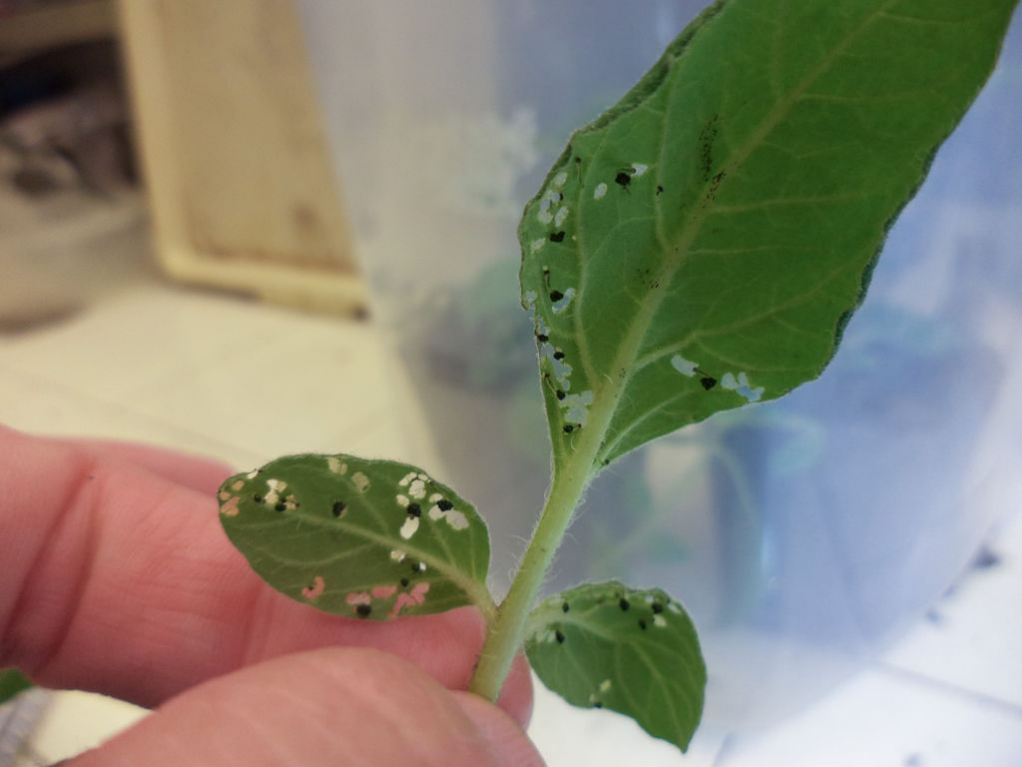
First and second instar larvae of *T. schrottkyi* with feeding marks on *S. commersonii*.

**Figure 4.**
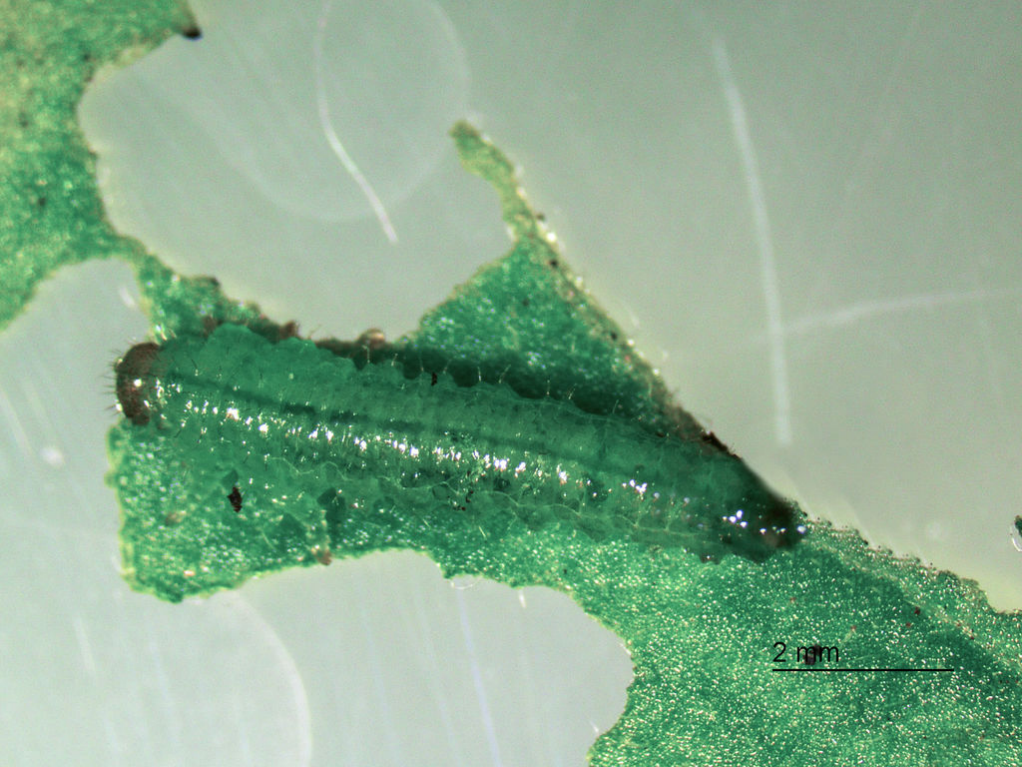
Later instar larva of *T. schrottkyi*.

**Figure 5.**
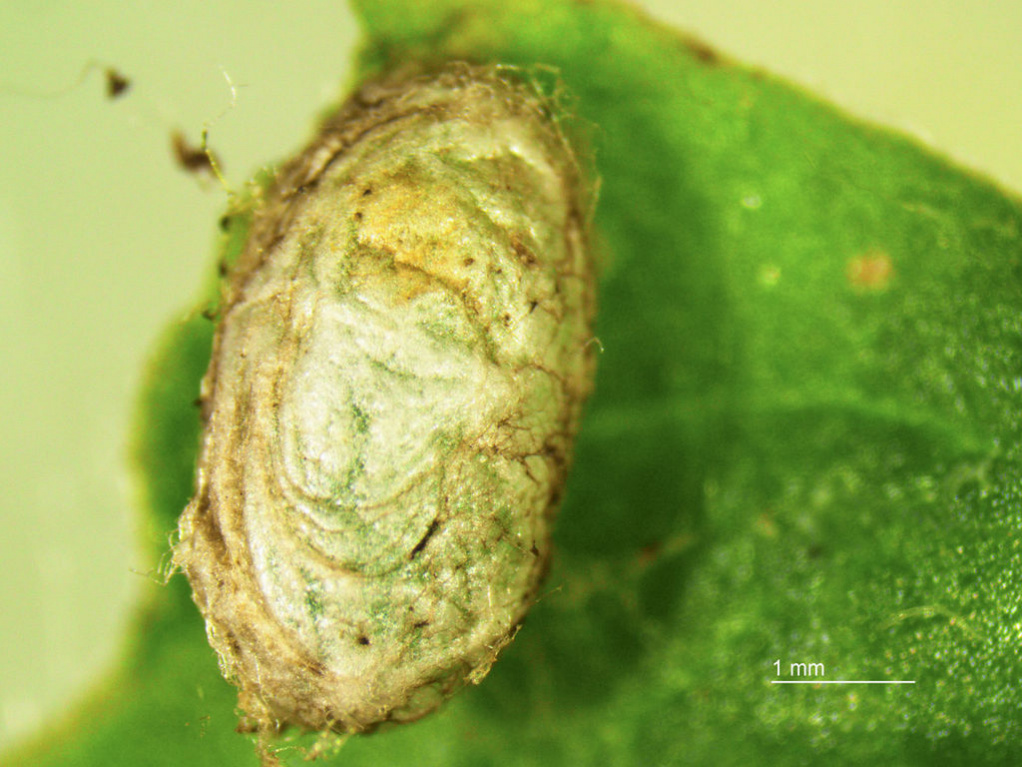
Cocoon of *T. schrottkyi*.

Between March and July *T. schrottkyi* presents several generations. Field temperature range measured during the sampling months was of 25 ± 4 °C (average maximum in March) and 5 ± 4 °C (average minimum in June) (mean ± SD) (INIA-Uruguay 2015). Under controlled laboratory conditions (21 ± 3 °C, 50 ± 10 % RH, 14:10 L:D regime), larvae collected as first and second instars (Figs 2, 3) and maintained on *S. commersonii* feeding on the leaves successfully completed their larval stage in less than 5 days and the prepupal and pupal stage to adulthood in aproximately 9 days (P. Altesor, unpublished) (Fig. 4). Mature larvae form a silk cocoon in the soil in which they pupate (Fig. 5), with female pupae roughly twice as large as male pupae (20.4 ± 1.1 mg, N = 14 and males: 9.3 ± 0.5 mg, N = 12 (mean ± SEM).

#### Biology

**Figure 6.**
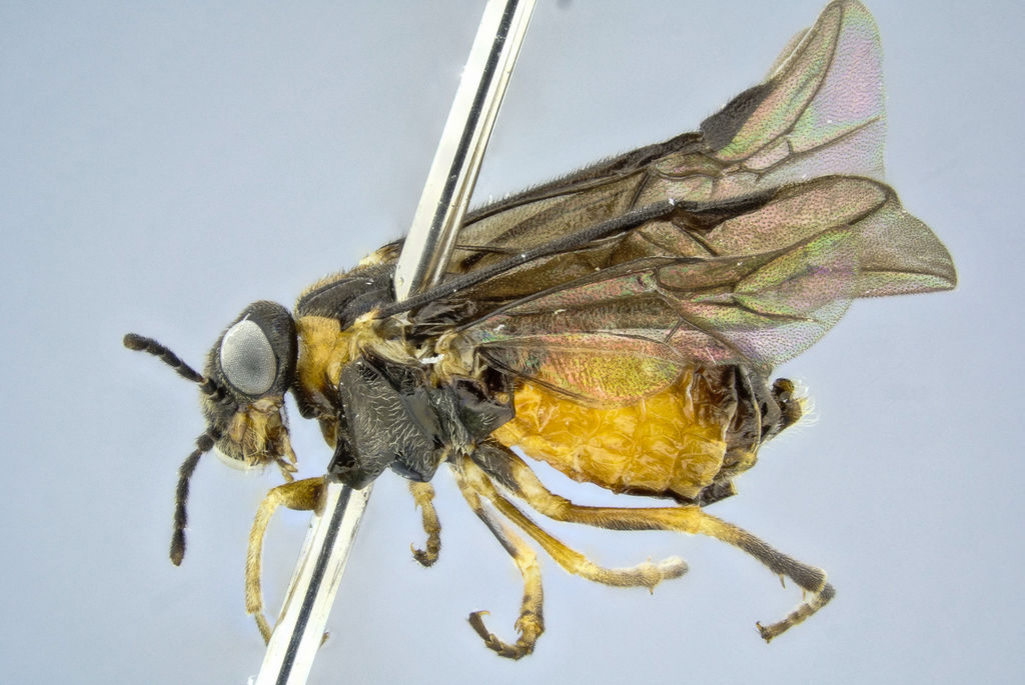
Female of *T. schrottkyi* in lateral view (BOLD sample ID: BC-ZSM-HYM-21584-E11).

**Figure 7.**
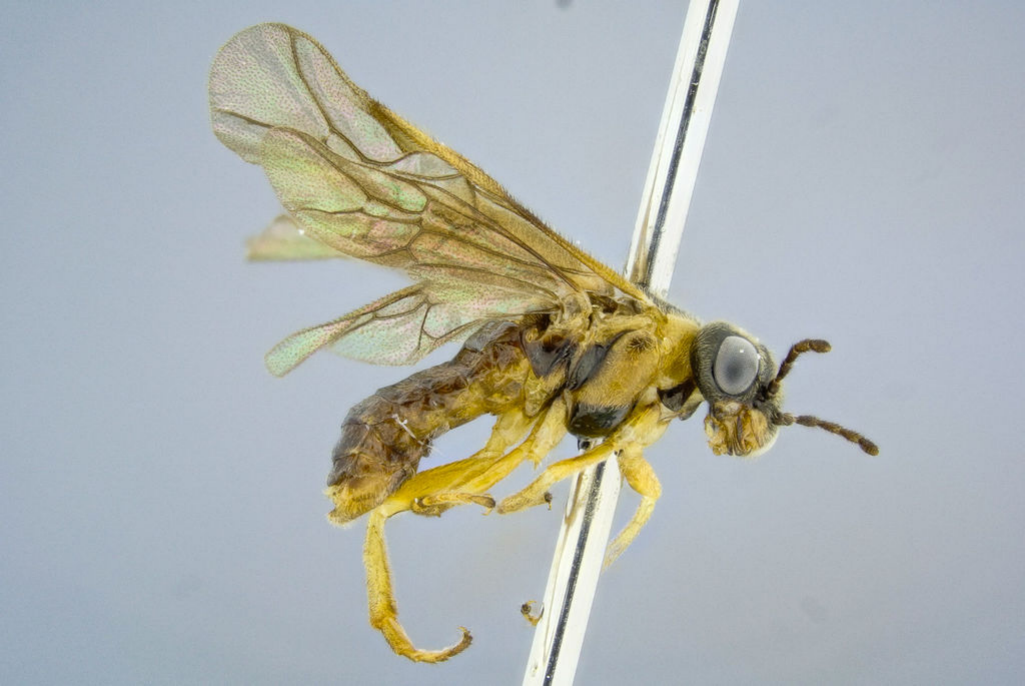
Male of *T. schrottkyi* (BOLD sample ID: BC-ZSM-HYM-21584-F01.

**Figure 8.**
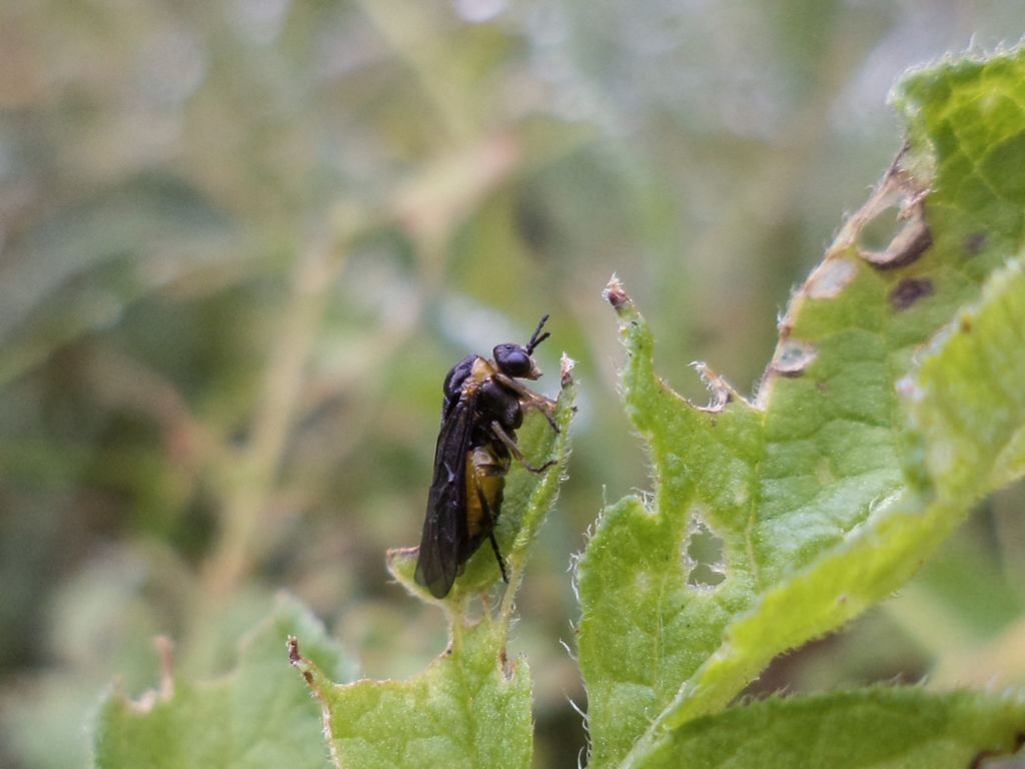
Female of *T. schrottkyi* on *S. commersonii* at INIA-Las Brujas field station.

Adults are sexually dimorphic, the female being larger than the male and with a different colour pattern. Compared to females, males have the thorax more extensively orange, and the abdomen black except more or less yellow orange laterally (in the female, the abdomen is orange except basally and apically more or less black) (Figs 6, 7, 8). Sexual dimorphism is common in the Pergidae, and often is expressed by differences in the antennal structure, color, and body size (Schmidt and Smith 2006).

**Figure 9.**
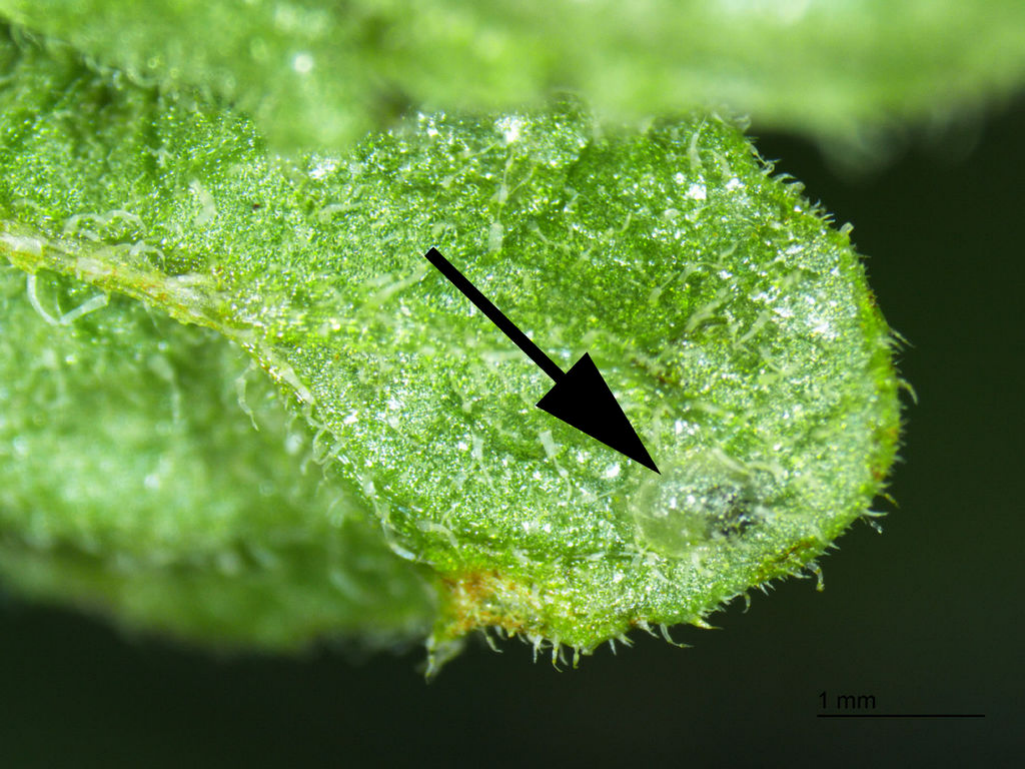
Egg of *T. schrottkyi* on *S. commersonii*.

Females lay the eggs individually in the leaf margin, into the leaf tissues as is typical of Symphyta (Smith 1990, Smith 1972, Weltz and Vilhelmsen 2014) (Fig. 9) (there are several eggs per leaf, but not clustered). In the laboratory, virgin females (24 - 48 h of age) laid eggs on *S. commersonii*, from which only male larvae emerged (arrhenotokous parthenogenesis).

## Discussion

 Three *Tequus* species are known to feed on *Solanum*, i.e. *Tequus munroi* (Smith) in Bolivia (Munro 1954), *Tequus willei* (Smith) in Peru (Wille 1943), and *Tequus ducra* (Smith) also in Peru (Arestegui 1976, Carrasco 1967, Ormachea and Galindo 1994). Possibly there is a fourth species, in Peru (García-Sinche and Catalán-Bazán 2011), but that species was not indentified and could be one of the three described ones. Biological studies exist only for two species, i.e. *Tequus ducra* (Carrasco 1967, Ormachea and Galindo 1994) and *Tequus* sp. (García-Sinche and Catalán-Bazán 2011). Both species produce at least three generations per year, feeding on cultivated potato. The eggs are laid into the leaf tissues on the underside, near the veins. The larvae pupate in the ground. Adults are sexually dimorphic, the females being larger than the males. *Tequus* sp. enter diapauses for six months, from April to October, as a prepupa in a silk cocoon. This diapausing period appears to be synchronised with the absence of the host plant and the dry season (García-Sinche and Catalán-Bazán 2011).

Among sawflies, the sequestration of defensive secondary metabolites derived from their host plants has been reported especially in Tenthredinidae and Pergidae (Opitz and Müller 2009, Morrow et al. 1976). In Pergidae, species of the Australian subfamily Perginae feed on *Eucalyptus* and related Myrtaceae. Larvae posess morphological adaptations on their mandibles to separate essential oils from nutritive plant matter, and store the oils in foregut diverticular pouches from where they are regurgitated for defensive purposes or at night without being disturbed, apparently as a mechanism to eliminate host-associated oils (Schmidt et al. 2000, Schmidt et al. 2010). In *T. schrottkyi*, preliminary tests with larvae facing the generalist predator *Schizocosa malitiosa* (Araneae, Lycosidae) resulted in the rejection of all larvae after contact, suggesting the presence of deterring substances. The host plant *S. commersonii* produces toxic glycoalkaloids, typical of some Solanaceae (Eich 2008), and ongoing studies focus on examining these plant metabolites as potential candidates for defensive substances used for defense in *T. schrottkyi*.

## Supplementary Material

XML Treatment for Tequus schrottkyi
